# The Regulatory Role of *α*-Ketoglutarate Metabolism in Macrophages

**DOI:** 10.1155/2021/5577577

**Published:** 2021-03-29

**Authors:** Shaojuan Liu, Jie Yang, Zhenfang Wu

**Affiliations:** College of Animal Science and National Engineering Research Center for Breeding Swine Industry, South China Agricultural University, Guangzhou, Guangdong 510642, China

## Abstract

Macrophages are multifunctional immune cells whose functions depend on polarizable phenotypes and the microenvironment. Macrophages have two phenotypes, including the M1 proinflammatory phenotype and the M2 anti-inflammatory phenotype, which play important roles in many inflammatory responses and diseases. *α*-Ketoglutarate is a key metabolite of the TCA cycle and can regulate the phenotype of macrophage polarization to exert anti-inflammatory effects in many inflammation-related diseases. In this review, we primarily elucidate the metabolism, regulatory mechanism, and perspectives of *α*-ketoglutarate on macrophages. The regulation of macrophage polarization by *α*-ketoglutarate may provide a promising target for the prevention and therapy of inflammatory diseases and is beneficial to animal health.

## 1. Introduction

Macrophages are an important part of the immune system and play vital roles in host defense and inflammation. Macrophages originate from monocytes released by the bone marrow, and they can migrate to different organs under natural or pathological conditions to form macrophages [1]. Under different pathological conditions, macrophages are polarized into two inflammatory phenotypes: the M1 proinflammatory phenotype and the M2 anti-inflammatory phenotype. The M1 phenotype is classically activated macrophages induced by various proinflammatory factors, such as lipopolysaccharide (LPS), interferon-*γ* (INF-*γ*), and tumor necrosis factor-*α* (TNF-*α*) [2–4]. M1 macrophages can excrete multiple proinflammatory cytokines such as interleukin-1 (IL-1), interleukin-12 (IL-12), and interleukin-23 (IL-23), and they can eliminate pathogens and activate adaptive immunity [5]. Conversely, M2 macrophages are alternatively activated macrophages induced by anti-inflammatory factors such as interleukin-4 (IL-4), glucocorticoids, and granulocyte colony factor (G-CSF) [6]. M2 macrophages exhibit anti-inflammatory effects in response to inflammation and produce many anti-inflammatory cytokines such as interleukin-10 (IL-10), transforming growth factor-*β* (TGF-*β*), and interleukin-4 (IL-4) [7–9], which could promote wound repair, fibrosis, and bone reconstruction [10]. The polarizable phenotype of M1 and M2 macrophages is a dynamic process that depends on the microenvironment and is regulated by a variety of intracellular signaling molecules and pathways. Macrophages are characterized by heterogeneity and plasticity and exhibit different functions due to signals in the local microenvironment. The functions of macrophages are affected by metabolites, inflammatory signals, oxygen tension, and cytokines [11–13]. Additionally, many signaling pathways can also regulate the polarization of macrophages, such as the Janus kinase/signal transducers and activators of transcription (JAK/STAT) signaling pathway, phosphatidylinositol 3′-kinase (PI3K)/Akt signaling pathway, c-Jun N-terminal protein kinase (JNK) signaling pathway, notch signaling pathway, and nuclear factor kappa-light-chain-enhancer of B cell (NF-*κ*B) signaling pathway [14–17]. Of note, cellular metabolism could bidirectionally regulate the functional response of macrophages, and the microenvironment composition of cellular metabolism plays a vital role in macrophage functions; in turn, cellular metabolites alter tissue homeostasis [18]. Furthermore, the balance between the M1 and M2 phenotypes plays an important role in the occurrence and development of diseases such as tuberculosis, tumors, and atherosclerosis and contributes to maintaining host homeostasis [19–21].

The metabolic characteristics of macrophages are different in the M1 and M2 phenotypes. The metabolism of M1 macrophages is characterized by strengthening the pentose phosphate pathway (PPP), increasing fatty acid oxidation (FAO), and improving anaerobic glycolysis while reducing the oxidative phosphorylation (OXPHOS) process and suppressing the tricarboxylic acid (TCA) cycle [22–24]. In contrast, M2 macrophage metabolism is mainly dependent on FAO and OXPHOS approaches to exert anti-inflammatory effects and repair tissue damage while decreasing glycolysis and the PPP process [25, 26]. All of these approaches could provide energy for macrophage metabolism and functional responses. Moreover, many studies have suggested that some amino acid metabolites play important roles in the prevention and treatment of certain inflammation-related diseases by inducing the polarization of macrophages and regulating macrophage functions. For example, glutamine metabolism could modulate macrophage polarization to prevent obesity and diabetes [27]. Likewise, inhibition of the accumulation and inflammatory signaling pathway of succinate could suppress M1 polarization to exert anti-inflammatory effects [13]. Notably, as an important metabolite of the TCA cycle and a precursor of glutamate and glutamine, *α*-ketoglutarate serves as an energy source and plays an important role in immunity, bone development, intestinal health, and the oxidative system [28–30]. Moreover, *α*-ketoglutarate suppresses M1 macrophage activation but promotes M2 macrophage activation to exhibit anti-inflammatory effects by mediating metabolic and epigenetic reprogramming [31]. In this review, we emphasize the regulatory role of *α*-ketoglutarate in macrophage polarization and provide a reference for the prevention and treatment of inflammatory diseases.

## 2. *α*-Ketoglutarate Metabolism in Macrophages

*α*-Ketoglutarate is a key intermediate in the TCA cycle and is generated from isocitrate by the oxidative decarboxylation of isocitrate dehydrogenase (IDH) and glutamate by the oxidative deamination of glutamate dehydrogenase (GDH). Then, *α*-ketoglutarate is metabolized into succinyl-CoA, catalyzed by *α*-ketoglutarate dehydrogenase (*α*-KGDH). Additionally, glutamine can be converted into *α*-ketoglutarate under the catalysis of GDH and glutaminase (GLS) via glutaminolysis. Using multiple reaction monitoring (MRM) to detect the targeted ^13^C-metabolic flux profiling of glucose and its intermediate metabolites in macrophages, glucose was shown to enter the cytoplasm and activate the TCA cycle and glycolysis with increasing intracellular and extracellular metabolites and selected enzyme levels in HIV-1 viral protein R- (Vpr-) induced macrophages [32]. It was observed that *α*-ketoglutarate was metabolized into glutamate and increased intracellular and extracellular glutamate release, which was further converted into glutamine in HIV-1-infected macrophages; in turn, glutamine metabolism promoted the accumulation of extracellular glutamate and *α*-ketoglutarate. Moreover, IDH2, as a crucial enzyme in the TCA cycle, catalyzes isocitrate into *α*-ketoglutarate. In LPS-induced lung inflammation, IDH2 could regulate *α*-ketoglutarate production to modulate the proinflammatory response mediated by NF-*κ*B [33]. Similarly, GDH-mediated *α*-ketoglutarate can produce energy in the TCA cycle but also inhibit activation of the inhibitor of nuclear factor kappa-B kinase *β* (IKK*β*), thus suppressing NF-*κ*B activation [34].

**Figure 1:**
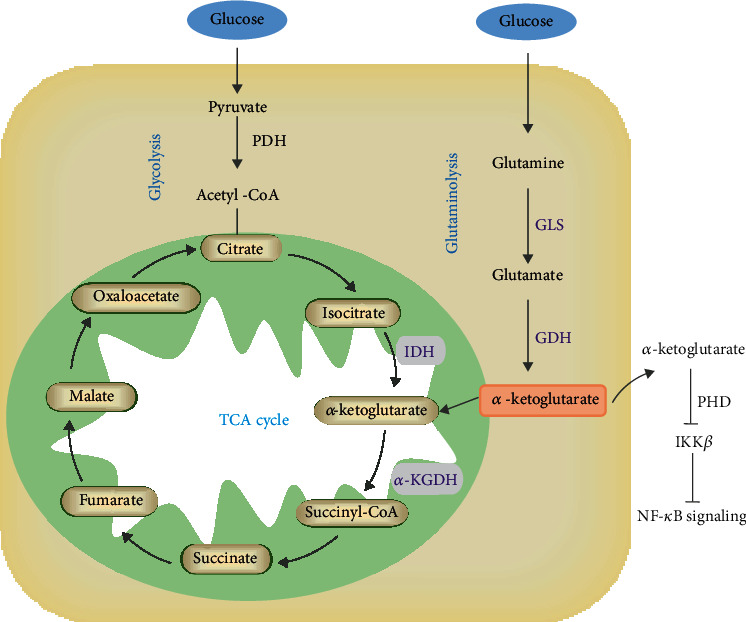
*α*-Ketoglutarate metabolism in macrophages. *α*-Ketoglutarate is generated from isocitrate by the oxidative decarboxylation of IDH in the TCA cycle or glucose via glycolysis. Additionally, glutamine could be converted into *α*-ketoglutarate under the catalysis of GDH and GLS via glutaminolysis. Then, *α*-ketoglutarate is metabolized into succinyl-CoA catalyzed by *α*-KGDH in the TCA cycle. In addition, GDH-mediated *α*-ketoglutarate can inhibit IKK*β* activation and block NF-*κ*B activation.

Collectively, *α*-ketoglutarate is derived from the oxidative decarboxylation of isocitrate in the TCA cycle and is produced from glutamine and glutamate metabolism or external sources, which play an important role in the polarization of macrophages by providing an energy source for damaged tissues (Figure 1). For example, glutamine metabolism increases the accumulation of *α*-ketoglutarate and glucose flux in the extracellular milieu of HIV-1-infected macrophages and HIV-1 Vpr-overexpressing macrophages via the glycolytic pathway and TCA cycle, indicating that *α*-ketoglutarate may be an energy resource for alleviating macrophage damage [32]. In addition, the isotope tracing method was used to investigate the pathways of glutamine metabolism in white spot syndrome virus (WSSV), and glutamine was found to be catabolized to glutamate by GLS and further converted to *α*-ketoglutarate by GDH to replenish the TCA cycle by the *α*-KGDH-mediated oxidative pathway and IDH-mediated reductive pathway [35]. Likewise, in HIV-1-infected or LPS-treated macrophages, *α*-ketoglutarate produced from glutamine metabolism by glutaminase 1 could promote extracellular vesicle release and regulate the inflammatory process [36].

## 3. *α*-Ketoglutarate Modulates Macrophage Polarization

**Figure 2:**
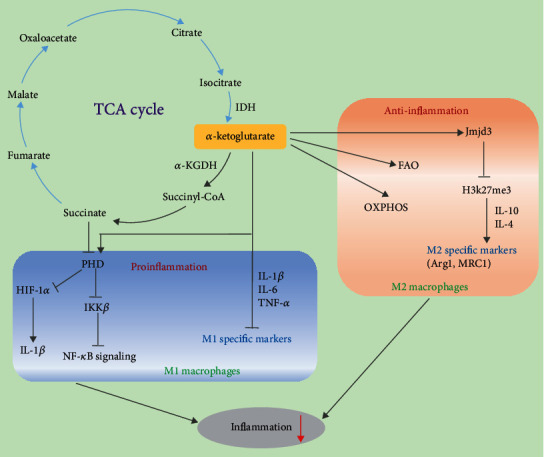
The regulatory mechanism of *α*-ketoglutarate on M1 and M2 polarization. In M1 macrophages, *α*-ketoglutarate inhibits M1 polarization by enhancing PHD activity to suppress IKK*β* activation and the NF-*κ*B pathway and inhibiting HIF-1*α* and IL-1*β* expression mediated downstream of succinate in the TCA cycle. In M2 macrophages, *α*-ketoglutarate generated by glutaminolysis is a checkpoint that regulates M2 metabolic reprogramming and the participation of FAO and OXPHOS in M2 macrophages. Additionally, *α*-ketoglutarate promotes M2 activation through the *α*-ketoglutarate-Jmjd3 pathway by suppressing H3K27me3 and increasing the expression of M2-specific markers.

*α*-Ketoglutarate can provide ATP for cell biological processes by activating the mammalian target of rapamycin complex (mTOR) signaling pathway and suppressing glutamine degradation in macrophage polarization [37, 38]. *α*-Ketoglutarate, as a product of glutamine metabolism, such as the glutamine-glutamate-*α*KG (Gln-Glu-*α*-KG) pathway, oxidative glutamine metabolism, and reductive carboxylation mediated by *α*-KGDH, IDH1, and IDH2, could refuel the TCA cycle and connect with the aerobic glycolysis and lipogenesis pathways in common with the PI3K-Akt-mTOR pathway. In M1-polarized MH-S cells, *α*-ketoglutarate notably decreased the protein expression of p70 ribosomal protein S6 kinase (P-p70S6K) in LPS-treated groups, but there was no evident change when *α*-ketoglutarate was added to LPS-rapamycin-treated groups. This result is consistent with the inhibition of LDH and the improvement of ATP production by *α*-ketoglutarate. Therefore, *α*-ketoglutarate may serve as an energy source to suppress the inflammatory response and then inhibit M1 macrophage activation induced by LPS [39]. Moreover, *α*-ketoglutarate could modulate the marker gene expression of M1 and M2 macrophages to alleviate inflammation (Figure 2). *α*-Ketoglutarate significantly decreased the serum levels of inflammatory cytokines (IL-6 and IL-12) and the expression of IL-1*β*, IL-6, and TNF-*α*, which are M1-specific markers, in lung tissues after 3 h LPS treatment, while it increased the anti-inflammatory expression of Arg1 and Mrc1, which are M2 marker genes. Similarly, *α*-ketoglutarate significantly facilitated peroxisome proliferator-activated receptor *γ* (PPAR*γ*) activation and the expression of Arg1 to promote M2 polarization compared to the IL-4-treated group of M2-polarized MH-S cells. In addition, *α*-ketoglutarate lowered the expression of M1 marker genes, increased the expression of M2 marker genes and IL-10, and suppressed NF-*κ*B signaling in LPS-induced rats [40]. Furthermore, *α*-ketoglutarate generated by glutaminolysis can act as a checkpoint to regulate M2 metabolic reprogramming and the participation of FAO in M2 macrophages. Supplementation with dimethyl-*α*-ketoglutarate (DM-*α*KG) could promote M2 activation through the *α*KG-Jmjd3 pathway by suppressing the demethylation of trimethylated histone H3 K27 (H3K27me3) and IL-4-induced genes. Notably, succinate, downstream of *α*-ketoglutarate, has been reported to increase the expression of the proinflammatory cytokine IL-1*β* mediated by HIF-1*α* in M1 macrophages, while the *α*-ketoglutarate/succinate ratio is subject to M1 and M2 macrophage polarization.

*α*-Ketoglutarate modulates the balance between M1 and M2 macrophage polarization by many means to relieve inflammation. In LPS-induced acute lung injury/acute respiratory distress syndrome (ALI/ARDS), *α*-ketoglutarate could inhibit M1 polarization by suppressing the mTORC1/p70S6K pathway and promote the M2 phenotype by enhancing PPAR*γ* nuclear translocation, which is conducive to preventing inflammatory diseases [39]. The addition of DM-*α*KG, a cell-permeable analog of *α*-ketoglutarate, restores the expression of the M2-specific gene Arg 1 and the ratio of *α*-ketoglutarate/succinate to promote M2 polarization in *P. gingivalis*-treated mouse bone marrow-derived macrophages (BMDMs) [41]. Cheng et al. suggested that DM-*α*KG produced by glutaminolysis could switch the polarization of M1 macrophages to M2 macrophages in Kupffer cells, which exerts anti-inflammatory effects by inhibiting NF-*κ*B activity and increasing the phosphorylation of glycogen synthase kinase 3*β* (p-GSK3*β*) and the expression of suppressor of cytokine signaling 1 (SOCS1) during the prevention and alleviation of hepatic ischemia-reperfusion injury (IRI) [40].

Metabolic and epigenetic remodeling play crucial roles in regulating macrophage reprogramming and phenotypic polarization. In IL-4- or LPS-treated BMDMs, *α*-ketoglutarate metabolized by glutamine and the Jmjd3-dependent pathway modulates M2 metabolic reprogramming, which serves as a regulator to participate in FAO and manipulates the ratio of *α*-ketoglutarate/succinate [31]. *α*-Ketoglutarate inhibits M1 polarization by intervening in the NF-*κ*B pathway to enhance prolyl hydroxylase (PHD) activity to suppress IKK*β* activation. Glutaminolysis-derived *α*-ketoglutarate is conducive to improving endotoxin tolerance in macrophages by modulating NF-*κ*B signaling and the Jmjd3-dependent pathway [31]. Mechanistically, melatonin increases *α*-ketoglutarate levels to promote M2 macrophage polarization and enhance TET-mediated DNA demethylation by increasing exosome secretion through the STAT3/NF-*κ*B signaling pathway in mouse adipocytes, which is beneficial to prevent and treat inflammatory diseases [31]. Additionally, the generation of *α*-ketoglutarate enhanced by Rspondin3-induced metabolism assists in catalyzing DNA hydroxymethylation via ten-eleven translocations (TETs) in lung injury, which acts as a cofactor for epigenetic reprogramming in macrophages to prevent inflammatory lung injury [42].

## 4. Regulatory Effects of *α*-Ketoglutarate on Inflammation-Related Diseases in Macrophages

As described above, macrophages have two polarized phenotypes, including the proinflammatory phenotype of M1 macrophages and the anti-inflammatory phenotype of M2 macrophages under different environmental stimuli, and they exhibit different physiological functions. M1 macrophages have a bactericidal function, secrete proinflammatory factors and regulatory factors, and engage in complement-mediated phagocytosis, which is part of the first line of innate immune system defense: phagocytosis, elimination of foreign pathogens, and activation of the T cell adaptive immune response [43]. However, excessive proinflammatory M1 induces an inflammatory response and has a role in many inflammatory diseases, such as atherosclerosis and severe acute pancreatitis. Studies have shown that a large number of proinflammatory mediators produced by M1 macrophages could aggravate lung injury and accelerate airway remodeling, resulting in the aggravation of asthma [44]. M2 macrophages could promote wound healing and fibrosis, repair tissue, and facilitate tumor growth [20, 45, 46]. Recent studies have suggested that certain metabolites can regulate the activity of epigenetic enzymes to modulate the polarization of macrophages through epigenetic mechanisms to affect the occurrence and development of inflammation-related diseases.

The intermediate metabolites of the TCA cycle could serve as energy sources to mediate the polarization of macrophages through epigenetic mechanisms and prevent certain metabolic diseases. *α*-Ketoglutarate is an important short-chain carboxylic acid molecule and a key intermediate in the TCA cycle that connects the key nodes of carbon-nitrogen metabolism in cells and provides carbon source materials and energy for cell growth and proliferation. Previous studies indicated that *α*-ketoglutarate could regulate the occurrence and development of certain inflammatory diseases by manipulating the polarization of macrophages. It has been reported that the administration of *α*-ketoglutarate could enhance beige/brown adipogenesis to reverse obesity by strengthening DNA demethylation [47, 48]. Similarly, a study found that melatonin could regulate the levels of cellular and exosomal *α*-ketoglutarate to enhance the polarization of M2 macrophages and TET-mediated DNA demethylation as a result of alleviating adipose inflammation in mice [49]. In addition, the number of M1 macrophages is increased, the levels of *α*-ketoglutarate and glutamine are decreased, and an accumulation of succinate has been observed in obesity and diabetes [27]. Therefore, *α*-ketoglutarate may be a promising target to prevent and treat obesity or diabetes. In LPS-induced lung injury, *α*-ketoglutarate could effectively promote the polarization of M2 macrophages and decrease inflammation to ameliorate lung damage via PPAR*γ* nuclear translocation and the mTORC1/p70S6K pathway [39]. Likewise, DM-*α*KG could inhibit NF-*κ*B activity and the secretion of proinflammatory cytokines by maintaining a higher ratio of M2/M1 polarization to relieve liver injury [40]. Thus, *α*-ketoglutarate may be a potential target on macrophages for the prevention and treatment of inflammation-related diseases.

## 5. Conclusion and Perspectives

Collectively, *α*-ketoglutarate is a key metabolite of the TCA cycle, which is metabolized in many ways, such as through the TCA cycle, glutaminolysis, and external sources. *α*-Ketoglutarate could serve as a cofactor to modulate the polarization of M1 and M2 macrophages and alleviate the inflammatory response and inflammation-related diseases. However, the current research results are not sufficient to fully explain the regulatory mechanism of *α*-ketoglutarate on macrophage polarization, and there is a need to further investigate its potential effects on macrophages. Of note, we summarized many studies on *α*-ketoglutarate and observed that most of them are related to intestinal function. Interestingly, many studies have demonstrated that intestinal macrophages are the first line of intestinal mucosal immunity and that they play a crucial role in intestinal homeostasis and numerous gastrointestinal diseases [50, 51]. Thus, we propose that the effects of *α*-ketoglutarate and their mechanisms on intestinal macrophages are worth investigating. Furthermore, we also discovered that macrophage polarization is associated with aging [52, 53]. *α*-Ketoglutarate can extend lifespan and postpone aging by regulating cell energy metabolism [54–56]. For instance, it was reported that *α*-ketoglutarate could ameliorate age-related osteoporosis by regulating histone methylation, reducing the expression of H3K9 m9e3 and H3K27me3, and increasing BMP signaling and Nanog [57]. Mechanistically, *α*-ketoglutarate mainly serves as an energy regulator by modulating ATP synthesis, limiting the energy utilization efficiency of nutrients, and maintaining a restricted diet state of organisms to ameliorate aging and age-related diseases through the mTOR pathway and AMPK signaling, and it is regarded as a potential antiaging agent [55, 58, 59]. Notably, a recent study indicated that the mechanism of extending longevity by *α*-ketoglutarate is related to a reduction of systemic inflammation and the elevation of IL-10 in aged female mice [54]. Therefore, in view of the above studies, we speculate whether *α*-ketoglutarate could extend lifespan and ameliorate aging or age-associated diseases by regulating the polarization of macrophages. However, it is not clear if the effect of *α*-ketoglutarate on longevity is applicable to humans, and the necessary levels of *α*-ketoglutarate to extend lifespan have not been quantified in animals, but these are areas worthy of further exploration.
